# Catalytic Gold Deposition for Ultrasensitive Optical Immunosensing of Prostate Specific Antigen

**DOI:** 10.3390/s20185287

**Published:** 2020-09-16

**Authors:** Laura Cid-Barrio, Jorge Ruiz Encinar, José Manuel Costa-Fernández

**Affiliations:** Department of Physical and Analytical Chemistry, University of Oviedo, Av. Julián Clavería 8, 33006 Oviedo, Spain; cidlaura@uniovi.es (L.C.-B.); ruizjorge@uniovi.es (J.R.E.)

**Keywords:** quantum dots, immunoassay, biomarker, nanotechnology, signal amplification

## Abstract

A major challenge in the development of bioanalytical methods is to achieve a rapid and robust quantification of disease biomarkers present at very low concentration levels in complex biological samples. An immunoassay platform is presented herein for ultrasensitive and fast detection of the prostate-specific antigen (PSA), a well-recognized cancer biomarker. A sandwich type immunosensor has been developed employing a detection antibody labeled with inorganic nanoparticles acting as tags for further indirect quantification of the analyte. The required high sensitivity is then achieved through a controlled gold deposition on the nanoparticle surface, carried out after completing the recognition step of the immunoassay, thus effectively amplifying the size of the nanoparticles from nm to µm range. Due to such an amplification procedure, quantification of the biomolecule could be carried out directly on the immunoassay plates using confocal microscopy for measurement of the reflected light produced by gold-enlarged nanostructures. The high specificity of the immunoassay was demonstrated with the addition of a major abundant protein in serum (albumin) at much higher concentrations. An extremely low detection limit for PSA quantification (LOD of 1.1 fg·mL^−1^ PSA) has been achieved. Such excellent LOD is 2–3 orders of magnitude lower than the clinically relevant PSA levels present in biological samples (4–10 ng·mL^−1^) and even to monitor eventual recurrence after clinical treatment of a prostate tumor (0.1 ng·mL^−1^). In fact, the broad dynamic range obtained (4 orders of magnitude) would allow the PSA quantification of diverse samples at very different relevant levels.

## 1. Introduction

One of the key goals of modern medicine is the identification and quantification of biological markers (biomarkers), which are key pieces of information that allow for the examination of normal biological processes, pathogenic processes, or pharmacologic responses to a therapeutic intervention. Particularly, absolute quantification of biomolecules present at very low concentration levels in biological samples is critically important in early medical diagnosis and evaluation of disease progression [1], resulting in a strong demand for highly selective bioanalytical approaches for the detection of biomarkers at very low concentration levels nowadays. In this regard, immunoassays based on the specific recognition of antigen–antibody have become a major bioanalytical tool when rapid and highly sensitive biomolecule quantification methods are required. In immunoassays, quantitation of the analyte depends on the reaction of an antigen (analyte) and an antibody. Traditionally, the detection antibodies used in biomarker quantification have been directly conjugated to organic fluorophores. However, the exponential growth experienced by nanotechnology during the last decades has produced a vast number of novel nanoparticles that are very promising to be used as labels in the development of improved immunoassays because of their unique optoelectronic properties, good biocompatibility, and high surface-to-volume ratio [2,3,4].

In fact, many different exciting luminescent nanoparticle (NP)-based immunoassays have been successfully developed so far [5,6]. However, the sensitivity of these assays are often insufficient to attain the extremely low concentration levels required to quantify biomarkers in complex biological matrices [5]. This is the case of the prostate-specific antigen (PSA), a serine protease well known as a prostate cancer biomarker. Total PSA concentration in healthy men serum is below 4 ng·mL^−1^, while an increase of serum levels above 10 ng·mL^−1^ indicates high probability of the disease [7,8]. Moreover, to monitor the eventual recurrence after a cancer treatment, PSA must be monitored at much lower concentration levels. In such cases, PSA levels have been defined as a rising from <0.1 ng·mL^−1^ (considered normal level) to a persistent >0.2 ng·mL^−1^, and this occurs in up 40% of men who are surgically treated [9]. In addition, recently PSA has been reported to be a potential biomarker for female breast cancer in early stages. Normal PSA concentration in women serum samples was found to be 1 pg·mL^−1^ [10], which is typically undetectable using conventional immunoassays. Therefore, the development of ultrasensitive absolute quantification of biomarker strategies is clearly needed in modern medicine.

To achieve such ultrasensitive detection, different signal amplification strategies based on NPs have been reported, but usually require complex and time-consuming steps and expensive reagents [11,12]. As an alternative to such complex methodologies, the sensitivity achieved in the immunoassays using NPs as labels can be drastically enhanced employing a simple and easily handled catalytic deposition of metals on the NP’s surface [13,14,15]. Based on such innovative amplification approaches, a highly sensitive immunoassay for PSA quantification has been developed based on the selective gold deposition on the quantum dot (QD) surface [16]. Despite this excellent sensitivity achieved using high-cost and time-consuming elemental mass spectrometry detection, such a strategy requires an additional acidic digestion step in order to obtain the corresponding dissolve metals from NPs. Such a step is usually time-consuming and often provides an incomplete digestion process, which could affect the quantitative results, since the biomarker concentration is indirectly obtained from the quantification of the metal dissolved [17]. The possibility of using a simpler and faster detection method and avoiding complex sample pretreatment or digestion steps would improve the applicability of these innovative approaches in clinical diagnosis. In this sense, previously reported studies focused on the imaging of different nanomaterials using optical detection [18,19], combined with size-amplified NPs obtained after catalytic metal deposition [15], open the doors to the possibility of carrying out a simple immunoassay strategy for quantification of low abundant biomarkers using optical detection carried out directly in the immunoassay plate.

Herein, we evaluated an ultrasensitive quantitative immunoplatform for biomarker determination, using PSA as the model analyte, based on an easy handling, and selective controlled catalytic deposition of gold on the surface of inorganic nanoparticles used as detection antibody tags. The amplified structures obtained, as consequence of the nanoparticle size-amplification process, were directly quantified simply using confocal microscopy in the same solid phase surface used for the immunoassay (i.e., microscope slides), thus avoiding the typical time-consuming digestion or isolation steps required for conventional elemental detection.

## 2. Materials and Methods

### 2.1. Reagents and Materials

#### 2.1.1. Reagents

All the experiments were carried out using analytical grade reagents used as received without any further purification. Deionized ultrapure water (18.2 MΩ·cm) was obtained with a PURE LAB flex3 (ELGA Labwater, High Wycombe, UK) and it was used throughout the present work.

For the synthesis of L-cysteine-capped Mn-ZnS QDs and citrate-capped AuNPs, zinc sulfate heptahydrate, manganese chloride tetrahydrate, L-cysteine hydrochloride monohydrate, and hydroxylamine were obtained from Merk (Darmstadt, Germany). Sodium sulfide nonahydrate, sodium hydroxide, lipoic acid, potassium tert-butoxide, bovine serum albumin (BSA), casein from bovine milk, sodium tetrachloroaurate (III) dehydrate (NaAuCl_4_), sodium citrate tribasic trihydrate, N(3-Dimethylaminopropyl)-N′-ethylcarbodiimide hydrochloride (EDC), N-hydroxysuccinimide (NHS), and Tween 20 were purchased from Sigma (Schnelldorf). Finally, rabbit polyclonal anti-PSA, mouse monoclonal anti-PSA, and prostate-specific antigen standard were obtained from Abcam (Cambridge, UK).

Poly-L-lysine surface-coated microscope slides (Electron Microscopy Sciences, Hatfield, PA, USA) with an adhesive press-to-seal silicon isolator to define wells (Grace bio-labs, Bend, Oregon, USA) were used for confocal microscopy detection.

#### 2.1.2. Instrumentation

UV–vis absorption spectra from the nanoparticles investigated as immunoreagents tags were obtained on a Genesys 10S Thermo Scientific Spectrophotometer (Thermo Scientific, Dreieich, Germany).

QD phosphorescent measurements were performed with a Varian Cary Eclipse Spectrometer (Varian Iberica, Madrid, Spain) equipped with a xenon discharge lamp (peak power equivalent to 75 W), a Czerny–Turner monochromator, and photomultiplier tube detector (Model R-298). The emission spectra were recorded upon excitation at 290 nm and using RTP conditions, with a delay time of 0.2 and a gate time of 5 ms.

A laser confocal microscope spectra Leica TCS-SP2-AOBS, with a 63-oil immersion objective, was used to acquire images of the amplified structures in microscope slides. A confocal microscope was used in reflection mode to visualize the larger structures obtained after the amplification procedure. A Helium-Neon 543 nm laser was employed to irradiate the corresponding microscope slides. Confocal image processing was carried out using Confocal Uniovi Image J software as explained in the following procedure section.

### 2.2. Procedures

QDs and AuNPs were selected as antibody labels for sensitive PSA quantification via a sandwich-type immunoassay.

#### 2.2.1. Synthesis of Cysteine-Capped Mn-ZnS QDs and Ligand Exchange Process

Colloidal water-soluble L-cysteine-capped Mn-ZnS QDs were synthesized following a previously reported protocol, using an Mn:Zn molar ratio of 3% [20]. A further surface QD functionalization step was required to control the bioconjugation process between QDs and antibodies. Surface coating was modified using dihydrolipoic acid (DHLA) following the procedure described elsewhere [21] with some modifications. DHLA agent is a bifunctional ligand with two anchoring points to bind the surface of nanoparticles, thus increasing the stability of the surface coating. First, 10 mg powder L-cysteine-capped QDs were mixed with 500 µL of dihydrolipoic acid. The reaction was performed for 2 h at 80 °C and left to complete overnight at room temperature with constant stirring. The purification process was carried out by precipitation of the resulting suspension with an excess of potassium tert-butoxide in 4 mL of methanol. The resulting solution was centrifuged at 5000 rpm for 5 min. Finally, nanoparticles were resuspended in water after a further purification step by filtration using 10 kDa Amicon ultracentrifugation filters.

#### 2.2.2. Synthesis of Gold Nanoparticles

Gold nanoparticles (AuNPs) were prepared following a procedure described elsewhere [22]. Briefly, 98 mL of Milli-Q water and 1 mL of 25 mM NaAuCl_4_ were placed in a flask and, then, heated under continuous stirring. Once the mixture was boiling, 1 mL of 33 mg·mL^−1^ sodium citrate tribasic solution was added quickly. The mixture was allowed to react for 10 min under boiling. Finally, AuNPs were purified by centrifugation at 7500× *g* for 30 min (×3) and they were resuspended in 0.01% Tween 20 (*v*/*v*).

#### 2.2.3. Bioconjugation Reactions

In order to obtain an appropriate bioconjugate between the PSA-antibody and QDs, EDC/NHS was used to form an amine bond between carboxylic groups from the DHLA ligands present on the surface of the QDs and amine residues from the mouse monoclonal anti-PSA antibody. The bioconjugation reaction was carried out in 10 mM pH 7.4 phosphate-buffered saline (PBS) 0.05% Tween 20 (*v*/*v*) for 2 h at room temperature with constant stirring using QD:Ab 3:1 and QD:EDC:NHS 1:1500:3000 molar ratios. During the first 15 min, QDs were mixed with EDC and NHS solutions in order to activate carboxylic groups, and then the corresponding antibody solution was added. Purification of the Ab-QD bioconjugate from the excess of reagents was carried out by ultrafiltration using a 100 kDa membrane filter. Experimental conditions used were 13,000 rpm, 10 min, and three cycles. Then, the bioconjugate was diluted in 10 mM PBS 0.05% Tween 20 and stored at 4 °C until its use. The corresponding emission spectra of the DHLA-capped Mn-ZnS QDs and the corresponding Ab-conjugates are given in Figure S1. As can be seen, labeling of antibody with QDs did not significantly affect the luminescent properties of the original QDs.

AuNP immunoprobes were prepared following a previously reported procedure with some few modifications [23]. Briefly, the AuNPs solution was adjusted to pH 8.5 using NaOH 1 M, and then the solution was incubated with a solution of monoclonal antibody anti-PSA using a molar ratio 1:1 (AuNPs:Ab) for 1 h at 4 °C. After incubation time, in order to cover the unconjugated NP surface, BSA was added to achieve a final concentration of 0.25% (*v*/*v*). The resulting mixture was incubated for additional 20 min. AuNPs-conjugated antibodies were separated from the unconjugated reagents by centrifugation at 12,000× *g* for 30 min at 4 °C, followed by resuspension in 10 mM PBS pH 7.4 containing 0.25% BSA (*w*/*v*) and 0.01% Tween 20 (*v*/*v*). The absorption spectra of AuNPs and their Ab-conjugates (AuNPs:Ab) are collected in Figure S2. As can be seen, optical properties of the AuNPs are preserved in the corresponding bioconjugates.

#### 2.2.4. Sandwich Type Immunoassay Format

**Figure 1:**
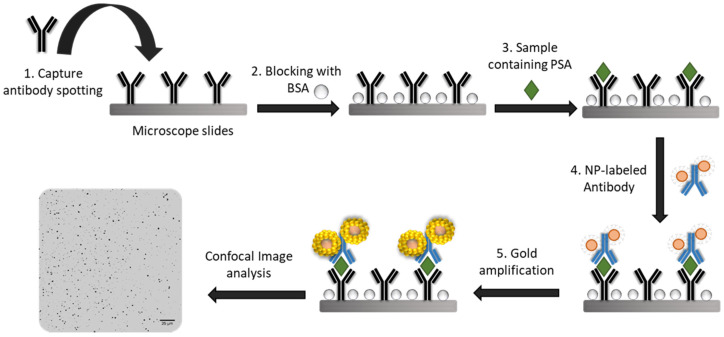
Schematic diagram of the nanoparticle (NP)-based immunoassay strategy for the detection of prostate-specific antigen (PSA) using catalytic deposition of gold on the surface of the NPs used as tags.

A schematic representation of the sandwich-type immunoassay is shown in Figure 1. In order to carry out confocal microscopy detection, microscopy glass slides were employed besides the commonly used ELISA plates and the corresponding wells were defined using an adhesive press-to-seal silicon isolator, which contained 8 wells. Poly-L-lysine microscopy slides were coated with 100 µL of a solution of 3 µg·mL^−1^ of the capture antibody (rabbit polyclonal anti-PSA) and incubated for 6 h at 37 °C. Then, the solution was removed from microscope slides and the surface was blocked with 150 µL 3% BSA overnight at 4 °C. After the blocking step, several washing steps were carried out using 150 µL 10 mM PBS pH 7.4 0.05% Tween 20 (three times). Then, 100 µL of the target samples were added to the corresponding wells and were incubated for 2 h at 37 °C. Another washing step was performed and 100 µL of 1 µg·mL^−1^, referred to as the antibody, of the bioconjugation solution (Ab:NPs) was deposited. After 2 h at 37 °C of incubation time, the solutions were removed, and a washing step was carried out again. Once the PSA immunoassay was completed, 100 µL the amplification solution, containing 1:1 0.5 mM NaAuCL_4_ and 5 mM hydroxylamine, was placed into the wells, and incubated for 15 min at 60 °C with constant stirring. A final washing step was performed using ultrapure water before confocal microscopy analysis.

It is worth mentioning that the total time expended for developing the complete immunoassay, without performing the final size-amplification step, was 22 h, which is in concordance with the time required for conventional sandwich-type immunoassays. The additional amplification step used in the described methodology only increased the total assay time in 15 min. This constitutes an important advantage over our previous protocol described by García–Cortés et al. [16] that required performing a quantitative particle digestion step of each immunoassay well and their corresponding dilution before the Au detection by ICP-MS, greatly increasing the total analysis time (ca. 6 h).

#### 2.2.5. Quantitative Image Processing of Confocal Microscopy Measurements

Digital images of the corresponding microscope slide wells were acquired after the immunoassay was carried out. In order to analyze a representative area from each well, 8 images were recorded in two different zones (4 images in each delimited zone). Confocal Uniovi Image J software was used for image processing. Reflection images were converted into binary forms, applying a proper threshold to classify all the pixels in two classes: Background (which corresponds to microscope slide surface) and foreground (which corresponds to enlarged particles). As a result, a binary image was obtained, where all pixels were classified as a_0_ = 1 (pixel value < threshold value), and a_1_ = 255 (pixel value > threshold value). The resulting binary image allowed us to distinguish between areas containing nanoparticles from background. Threshold value was selected in order to ensure that all the particles were properly computed and, then, applied to all the images acquired in the same experiment. Once the binary images were obtained, the Confocal Image J algorithm could be applied to count all the particles of a particular shape. We did not impose any restrictions on shape because the amplified particle detected could present different sizes, depending on the NPs used as catalytic seeds (i.e., AuNPs or Mn-ZnS QDs), and amplification conditions employed in each experiment. As a result of the particle analysis, the total particle number was calculated in each image. Finally, the results obtained for all the images acquired in the same delimited well zone were added in order to obtain a representative particle number of each zone.

## 3. Results and Discussion

### 3.1. Gold Deposition on the Surface of the Mn-ZnS QDs and AuNPS Acting as Catalytic Seeds

Metallic deposition on the surface of different nanoparticles and their consequent size amplification and further visualization by microscopic techniques have been already reported [24]. In a recent study, the controlled catalytic deposition of Au(III) ions was investigated in order to enhance the scattering light produced for AuNPs to detect low abundant target in transparent tissues using confocal microscopy [15]. The catalytic deposition of silver on CdSe/ZnS QDs or on AuNPs [23] have been also reported for quantitative purposes. Silver amplification has been demonstrated to be a simple and fast process but with low reproducibility owing to the difficulty in controlling the kinetic process that occurs during the amplification step [16]. In this regard, gold amplification on the surface of different NPs has also been evaluated [23,25,26]: Unlike silver amplification, gold deposition was demonstrated to be more effective and reproducible [16].

Here, we evaluated the possibility to perform the amplification process based on the controlled catalytic gold deposition on the surface of two different NPs with different nature and coating: Citrate-capped AuNPs and DHLA-capped Mn-ZnS QDs.

First, we determined whether the amplification process produced larger structures, and the possibility to be detected in the same solid phase platform where the complete immunoassay was performed (poly-*L*-Lysine-coated microscope slides). For this purpose, a sandwich-type immunoassay was performed for 100 pg·mL^−1^ PSA detection (a blank assay was also performed) using both types of nanoparticles as antibody labels, in order to determine if a selective deposition of metallic gold was produced on each NP surface. The amplification solution used was NaAuCl_4_, as the gold (Au (III)) precursor with hydroxylamine as the reducing agent, at final concentrations of 0.5 and 10 mM, respectively.

**Figure 2:**
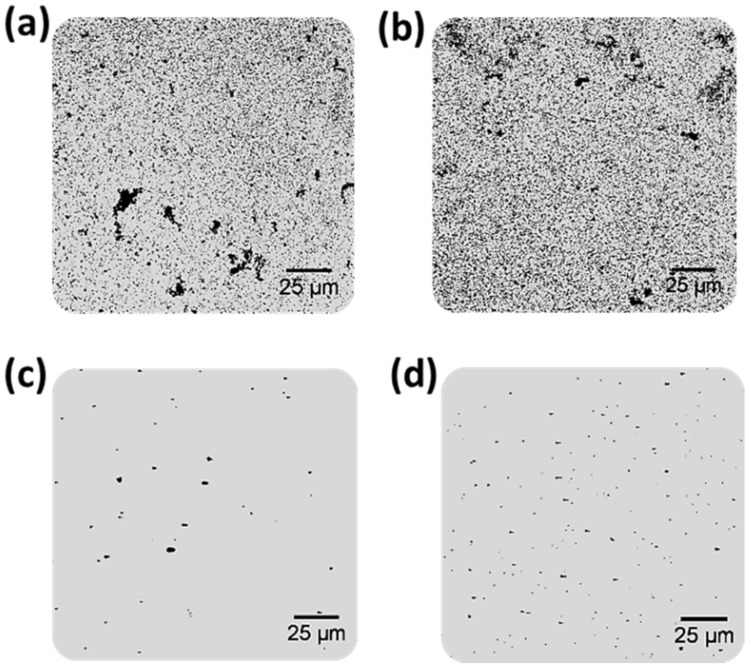
Confocal microscopy images of small areas (160 × 160 µm^2^) of the microscopy slide wells obtained after the immunoassay followed by gold deposition on the antibody labels (Mn-ZnS quantum dots (QDs) and gold nanoparticles (AuNPs)) at different PSA concentrations. (**a**,**b**) Amplified AuNPs, 0 and 100 pg·mL^−1^ PSA, respectively; (**c**,**d**) amplified QDs, 0 and 100 pg·mL^−1^ PSA, respectively.

Gold-enlarged structures were obtained and monitored by reflection confocal microscopy, making use of the capability of metallic structures to reflect the laser light employed to irradiate the microscope slide. The resulting images of the corresponding wells are collected in Figure 2. It is worth mentioning that the gold amplification procedure is produced after performing the complete PSA immunoassay (see Figure 1). Therefore, the recognition capabilities of the corresponding antibody were not compromised by steric effects due to the use of larger nanostructures.

**Figure 3:**
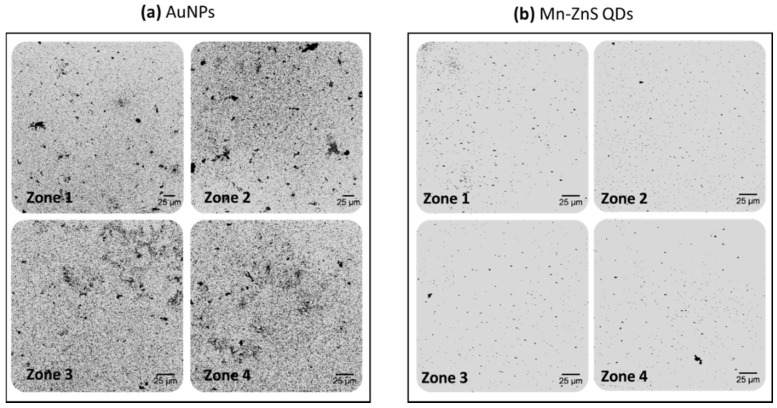
Confocal microscopy images of small areas (230 × 230 µm^2^) of the microscope slide wells obtained after the immunoassay followed by gold deposition using 10 pg·mL^−1^ PSA. The images were acquired from different zones of the same microscopy slide wells for each nanoparticle used as catalytic seeds (**a**) amplified AuNPs and (**b**) amplified Mn-ZnS QDs.

As expected, size-enhanced nanostructures were produced, resulting in an increase in the sizes of both nanomaterials used as antibody tags acting as catalytic seeds (AuNPs and QDs). However, different behavior was observed depending on the catalytic seed. Gold grew faster onto the AuNP surface, producing an almost instantaneous formation of AuNP aggregates on the microscope slides, which could be directly observed due to the blue-color darkening of the wells observed after the amplification step. Such visual amplification was easily observed using reflection confocal microscopy. Figure 2a,b shows an uncontrolled gold amplification produced on the AuNP surface even for a blank solution without PSA (Figure 2a). Additionally, the number and particle size obtained along all the well surfaces were very heterogeneous. This fact can be easily observed in Figure 3, where images from different zones of the same well were collected. Furthermore, the computation of the number of NPs in each image can be calculated, resulting in a poor reproducibility between the total number of NPs computed in the different images obtained from the same well (85% RSD).

In contrast, for QD amplification, lower background signals were obtained after the amplification of a blank solution (in the absence of PSA, Figure 2c). As expected, an increment on the number of size-amplified antibody tags were observed when samples with higher PSA concentrations were analyzed (100 pg·mL^−1^ PSA, Figure 2d). Gold amplification of the QDs used as antibody tags were more homogeneous (Figure 3b). Similarly to AuNPs, the reproducibility of gold amplification along the well was computed, resulting in a dispersion between different images recorded from the same well of 17%. These findings seem to indicate that the use of Mn-ZnS QDs as catalytic seeds offers better analytical features for PSA immunoassay by gold deposition and reflection confocal microscopy detection.

**Figure 4:**
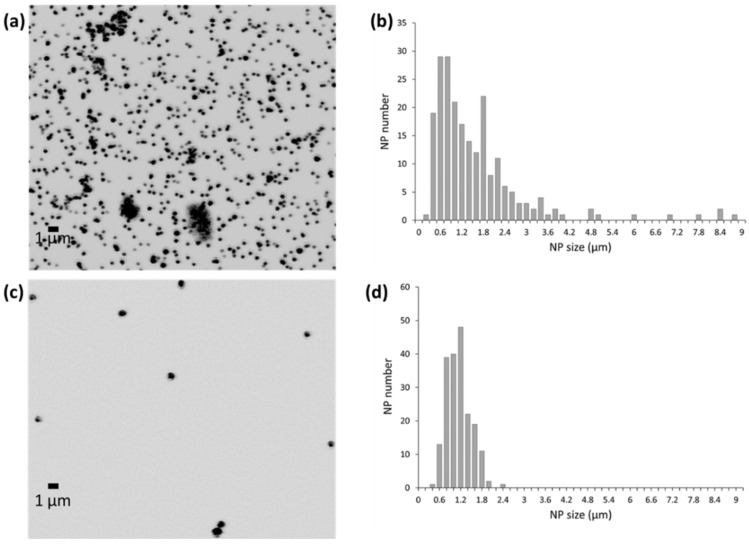
Confocal microscopy images for amplified (**a**) AuNPs and (**c**) Mn-ZnS QDs after performing the complete PSA immunoassay and the catalytic gold deposition. The corresponding size distribution obtained for the amplified nanostructures is also shown for (**b**) AuNPs and (**d**) Mn-ZnS QDs (*n* = 250 particles).

In fact, the enhanced NP size can be easily computed by comparing the HR-TEM images (collected in Figure S3) obtained for each nanoparticle and the corresponding confocal images obtained after the complete PSA immunoassay was performed. As can be seen in Figure 4a, the final size of AuNPs increases from 21 ± 1 nm to 1.5 ± 1.1 µm. In the case of Mn-ZnS QDs (3.6 ± 0.2 nm), the final size of amplified structures observed was 1.2 ± 0.3 µm. The dispersion observed in the size distribution of amplified AuNPs, 73%, (Figure 4b) was worse that those obtained for amplified Mn-ZnS QDs, 17% (Figure 4d).

In brief, we tested the catalytic potential of the Mn-doped ZnS QDs for gold deposition. The formation of the gold-enlarged nanostructures was confirmed by electron microscopy and confocal microscopy detection (an increase in the QD nanoparticle diameter from a few nanometers to hundreds of nanometers was observed, see Figure 4 and Figure S3), demonstrating that an autocatalytic reaction occurs in which metallic gold is deposited on the surface of the QDs, acting as nucleation sites. Moreover, formation of the gold-enlarged structures takes place during the first 15 min upon addition of the gold reagents. After that time, production of gold-enhanced structures is stabilized and does not proceed further. It must be highlighted that, in contrast to the rapid microstructure development observed when QDs were present, no significant gold-enlarged structures were observed in the absence of the QDs. Therefore, it was proven that QDs were actually required for gold reduction deposition. All such evidences indicated that the catalytic effect of QDs on Au(III) ion reduction deposition was highly efficient.

### 3.2. Optimization of the QD-Size Amplification Procedure

**Figure 5:**
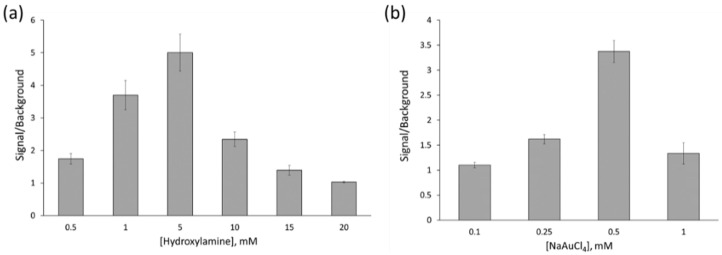
Au-amplified Mn-ZnS QD number measured (signal to background) after performing the PSA immunoassay using different amplification conditions, (**a**) hydroxylamine concentration (reducing agent) and (**b**) NaAuCl_4_ concentration, while keeping constant the rest of parameters. Error bars correspond to 1SD.

A key parameter in this procedure was the control of the production of size-amplified nanostructures. In order to achieve a highly sensitive and reproducible quantification, some relevant parameters had to be evaluated to obtain homogeneous gold deposition over the nanoparticle surface after the immunoassay. First, the concentration of solutions used for Au amplification (Au^3+^ salt and reducing agent) were optimized. For this purpose, samples containing 0 and 10 pg·mL^−1^ of PSA were analyzed. Different hydroxylamine concentrations (Figure 5a) were tested while keeping a constant NaAuCl_4_ concentration (0.5 mM). Then, different NaAuCl_4_ concentrations (Figure 5b) were assayed while keeping constant the reducing agent concentration (hydroxylamine, 5 mM). The corresponding images acquired in each well, and used for total particle area computations, are given in Figures S4 and S5 for hydroxylamine and NaAuCl_4_ optimization, respectively. The images acquired in each well were processed following the procedure previously described using the Confocal Uniovi Image J Software, allowing us to compute the total particle number which is presented in the corresponding wells. Results revealed that high amplification was observed when the concentration of the precursor solutions were increased. However, higher background signals were obtained for higher concentrations, resulting in an indistinguishable signal between background and PSA solution when 20 mM for hydroxylamine and 1 mM for NaAuCl_4_ were used. The ratio of the total particle number obtained for the PSA sample and blank solution was translated into signal-to-background (represented in Figure 5), which could be used as a diagnosis parameter. In fact, the best results of the S/B ratio for the optimization conditions were obtained using 0.5 NaAuCl_4_ and 5 mM hydroxylamine (see Figure 5a,b).

Additionally, different amplification times were assayed (10, 15, 20, and 30 min) after the complete immunoassay was performed using again a blank solution and a doped PSA sample. The resulting images are collected in Figure S6. As expected, the particle area obtained in each well increased when ensuring higher amplification times. Unfortunately, an increase in the background signal with amplification time was observed as well, as a consequence of the amplification of QDs:Ab bioconjugate unspecified adsorbed onto the microscope slides surface. In fact, for 30 min of amplification time, signals obtained for wells containing a control sample (without PSA) and doped PSA sample were indistinguishable. Similar results were obtained in absolute signals for 15, 20, and 30 min for wells containing PSA. However, as shown in Figure S8, the best signal-to-background ratio was provided for 15 min of amplification time, which was the final chosen time.

### 3.3. Confocal Microscopy Measurement of the Gold Amplification for Development of a PSA Immunoassay

In order to investigate the analytical performance of the developed immunoplatform, a sandwich-type immunoassay using an additional amplification step based on catalytic deposition of gold onto the Mn-ZnS QD surface was performed under optimal conditions, following the protocol described in the procedure section (Figure 1). In order to assess the analytical performance of the purposed gold-amplified immunoassay, increasing PSA concentrations (ranging 0.01–100 pg·mL^−1^) in 10 mM PBS (pH 7.4) were prepared to construct a dose–curve response. After performing the complete immunoassay and the gold amplification step, the obtained gold-enlarged QD structures were visualized by reflection confocal microscopy.

**Figure 6:**
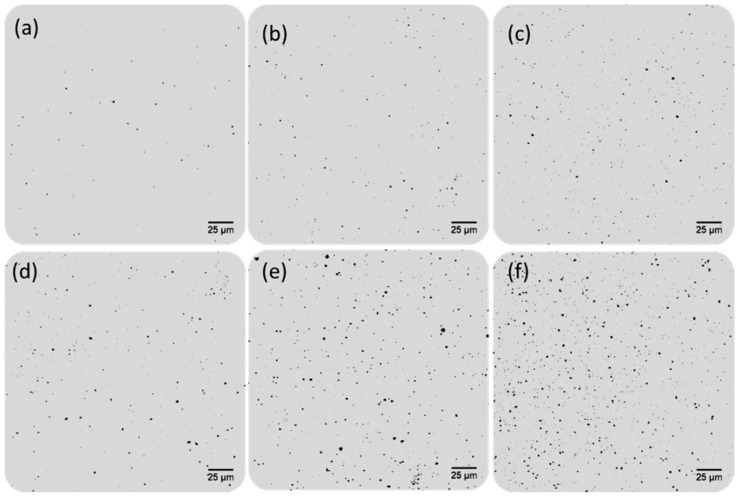
Confocal microscopy images of small areas (230 × 230 µm^2^) of the microscope slide wells obtained after the PSA QD-based immunoassay, followed by gold deposition on the Mn-ZnS QD tags at different PSA concentrations: (**a**) 0 PSA, (**b**) 0.01 PSA, (**c**) 0.1 PSA, (**d**) 1 PSA, (**e**) 10 PSA, and (**f**) 100 pg·mL^−1^ PSA.

**Figure 7:**
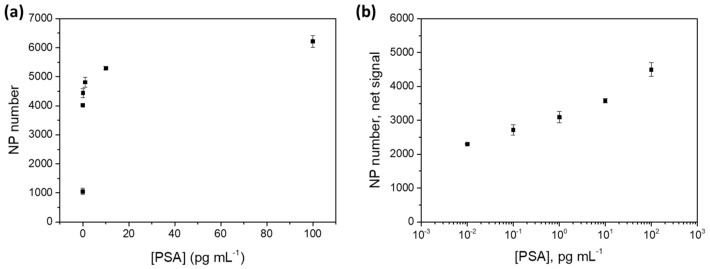
(**a**) Immunoassay response curve obtained after image processing of gold-amplified Mn-ZnS QD confocal microscopy images at the different PSA concentrations collected in Figure 6. (**b**) Immunoassay response curve linearized by applying Ln to the *x*-axis. Error bars correspond to 1SD of the two different zones measured in the same well. The corresponding calibration equation is: 228.07 Ln [PSA] + 3237.4; r^2^ = 0.966.

Figure 6 shows the images resulting from gold-amplified structures with increased PSA concentrations. The displayed spots observed in Figure 6 demonstrate, as expected considering that there was a sandwich-type format, that the higher the PSA concentrations, the more particles absorbing the laser light are observed. This method could then be used for PSA quantification since the optical signal is directly related with PSA concentration. In order to obtain a representative area of each well, 8 images were recorded in different zones of the same well and, then, these images were properly processed as described in the procedure section. Then, the number of Au-enlarged QD nanostructures in each image was computed. A typical dose response immunoassay curve was built with a lengthy part exhibiting a linear response between the analytical signal and the logarithm of the PSA concentration four orders of magnitude (see Figure 7). The limit of detection (LOD) was calculated according to the IUPAC protocol. This value was estimated as the concentration of PSA which produced an analytical signal equal to the mean of blank signal (*n* = 4) plus 3 times its standard deviation. Table S1 in the supplementary information section collects the data used to calculate the LOD, including values of slope, intercept, and regression coefficient of the calibration plot, as well as the blank signal value and its standard deviation. As a result, an LOD of 1.1 fg·mL^−1^ PSA was obtained. Such an extremely low LOD indicates that the proposed approach is particularly useful for ultrasensitive PSA quantification involving effectiveness of medical treatment, since the PSA levels narrowed to 0.1 ng·mL^−1^ in male serum samples after a prostatectomy [9]. In fact, such an excellent LOD could be used not only for the diagnosis and monitoring of the eventual recurrence of prostate cancer in men, but also for detection of PSA levels in women serum samples where PSA levels narrowed to 1 pg·mL^−1^ in serum samples were found [10]. It is worth noting that a similar sandwich-type immunoassay for PSA determination has been previously evaluated using the native phosphorescent emission of Mn-ZnS QDs (before the catalytic gold deposition) as the analytical signal. The detection limit obtained using RTP detection was of 17 pg·mL^−1^ PSA [27]. In contrast, the catalytic gold deposition procedure here described resulted in an improvement in the detection limit for PSA quantification of 4 orders of magnitude (1.1 fg·mL^−1^ PSA). This is low enough to face the quantification of relevant PSA levels in women sera, as recently PSA was demonstrated to be a potential biomarker for breast cancer at very low concentration levels (1 pg·mL^−1^ PSA) [10]. Table 1 summarizes some of the multiple bioanalytical methods designed for PSA quantification in clinical samples. The method here purposed is one of the most sensitive methods so far for PSA quantification in biological samples, with a broad dynamic range exceeding 4 orders of magnitude, based on a simple strategy. In the table, there are some other strategies that also provide excellent detection limits but require expensive instrumentation [16,28] and long analysis time [29]. It could be expected that the direct use of higher-size microparticles as antibody labels could be an alternative to the here-proposed methodology, without the need of an amplification step. However, the high affinity of microparticles for biomolecules could result in undesirable biomolecule unspecific adsorptions and poorer LODs.

To assess the applicability of the sandwich immunoplatform described for real sample analysis, the influence of another possible coexistent species in the real samples was evaluated. Since the most common samples employed for PSA determination in clinical diagnosis are serum samples which contain a higher amount of proteins, the determination of total PSA levels was evaluated in a sample containing a high amount of bovine serum albumin (BSA). For that purpose, synthetic samples containing PSA at concentration levels of 0 and 10 pg·mL^−1^ in the presence of a high protein content (BSA 1%) were analyzed following the complete immunoassay procedure. Then, the total amount of PSA present in such synthetic samples were computed using the dose response curve obtained as previously described. Images acquired from different positions of the corresponding wells are given in Figure S8. The presence of an interfered substance (BSA) in a very high content did not induce enhancement or attenuation of the signal and did not produce detectable negative effects in the formation of amplified gold structures. Moreover, after applying the corresponding calibration curve (Figure 7), the results obtained for the different well zones of the spiked sample provided a PSA level of 11.9 ± 0.8 pg·mL^−1^ (that is quantitative recovery of 119 ± 9%). Such results validate the proposed quantitative methodology and prove its potential to quantify PSA in clinically relevant samples.

**Figure 8:**
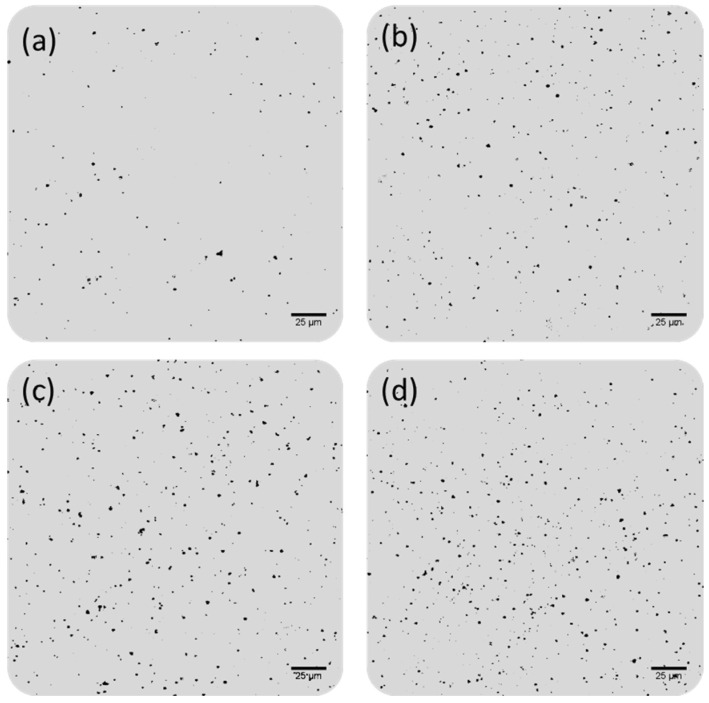
Confocal microscopy images of small areas (230 × 230 µm^2^) of the microscope slide wells obtained after the PSA QD-based immunoassay, followed by gold deposition on the Mn-ZnS QD tags at different PSA concentrations: (**a**) 0, (**b**) 5, (**c**) 10, and (**d**) 20 pg·mL^−1^ PSA used for methodology validation.

Finally, in order to validate the proposed methodology as a potential sensor for clinical diagnosis of prostate cancer, PSA content was determined in three samples containing close concentration levels of PSA: 5, 10, and 20 pg·mL^−1^ PSA. The corresponding images are collected in Figure 8. The experimentally determined PSA concentrations are given in Table 2 and were very close to the theoretical values in all the samples assayed: 5.3 ± 0.4, 10.3 ± 0.2, and 18.6 ± 0.8 pg·mL^−1^ PSA (quantitative recoveries ranging from 93 to 105%). Therefore a simply dilution 1:1000 of the serum samples would bring the clinical relevant concentration range (low ng·mL^−1^ PSA) to the middle of the calibration range (low pg·mL^−1^ PSA) where we have already clearly demonstrated that we were able to discriminate among close concentration levels and, therefore, to provide a reliable classification of the samples in the healthy and diseased.

## 4. Conclusions

A sandwich-type optical immunoassay was developed to quantify the protein biomarker PSA with an extremely high sensitivity after a simple sample readout performed directly over immunoassay plates. To achieve the desired ultrahigh sensitivity, a signal amplification step, consisting of a controlled homogeneous gold catalytic deposition on the surface of Mn-ZnS QDs used as tags of the detection antibody, was incorporated just before the detection step. The huge nanoparticle size enhancement of the QDs used as antibody tags by the controlled gold deposition performed after the immunoassay, followed by an optical detection of the microstructures, allowed for the development of an extremely sensitive immunoassay for PSA detection (LOD in the fg·mL^−1^ range), enabling the determination of relevant concentration levels of PSA in female sera. Although the dynamic range is not wide enough to allow simultaneous analysis of samples at higher concentration levels of clinical interest for male disease diagnostics (0.1–0.2 ng·mL^−1^), it is worth noting that the method can also be applied for such determinations just by performing a simple dilution of the male serum samples before the analysis.

The excellent sensitivity of the here-developed immunosensor, together with its high specificity and robustness, would enable the sensitive detection of many other biomarkers in routine clinical applications based on a simple and rapid optical detection, only requiring the selection of appropriate specific capture and recognition antibodies.

Recent developments in highly efficient smartphone-based portable optical devices make them convenient alternatives to confocal microscopy in order to perform the here proposed immunoassay in an efficient manner for daily applications. In fact, smartphone devices with a resolution of 1 µm or even lower values (0.8 µm), have been already developed [35] and have, therefore, the potential for the detection of the size-amplified nanoparticles produced as a consequence of the catalytic gold deposition presented herein.

## Figures and Tables

**Table 1:** Analytical performance characteristics of different biosensors for ultrasensitive PSA detection.

Analytical Label	Amplification Step	Detection Technique	Biosensor Format	LOD pg·mL^−1^	Dynamic Range	Ref
HRP	Enzymatic	Absorbance	Sandwich immunosensor	400	1–50 ng·mL^−1^	[30]
Eu, Sm, and magnetic beads	-	Time-resolved fluoroimmunoassay	Homogeneous sandwich immunosensor	50	0.5–100 ng·mL^−1^	[31]
Mn-ZnS QDs	Nanoparticle-enhanced sensitivity	Phosphorescence	Sandwich immunosensor	17	0.05–240 ng·mL^−1^	[27]
AuNPs	Nanoparticle-enhanced sensitivity	Surface plasmon resonance (SPR)	Colorimetric immunosensor	9	0.01–20 ng·mL^−1^	[32]
HRP	Enzyme	Amperometric	Sandwich immunosensor	1	0.002–10 ng·mL^−1^	[33]
Graphene oxide QD-Ag core-shell and magnetic beads	Nanoparticle-enhanced sensitivity	Fluorescence	Sandwich immunosensor	0.3	0.001–20 ng·mL^−1^	[34]
Au@AgNPs	Nanoparticle enhanced sensitivity	Surface-enhanced Raman scattering (SERS)	Aptasensor	2 × 10^−4^	0.3–16 pg·mL^−1^	[28]
AuNPs and CuNPs	Rolling circle amplification and cascade signal amplification	Voltamperometric	Aptasensor	2 × 10^−5^	0.05–500 pg·mL^−1^	[29]
Gold-amplified Mn-ZnS QDs	Catalytic gold deposition	ICP-MS (Au)	Sandwich immunosensor	26 × 10^−6^	1 pg–10 ng·mL^−1^	[16]
Gold-amplified Mn-ZnS QDs	Catalytic gold deposition	Reflection confocal microscopy	Sandwich immunosensor	3.5 × 10^−3^	0.01–100 pg·mL^−1^	This work

**Table 2:** PSA quantification and recovery for samples containing close PSA concentrations. Uncertainty associated corresponds to 1SD (*n* = 2 replicates).

Theoretical PSA Concentration	Experimental PSA Concentration	% Recovery
5 pg·mL^−1^	5.3 ± 0.4 pg·mL^−1^	105%
10 pg·mL^−1^	10.3 ± 0.2 pg·mL^−1^	103%
20 pg·mL^−1^	18.6 ± 0.8 pg·mL^−1^	93%

## Supplementary Materials

The following are available online at https://www.mdpi.com/1424-8220/20/18/5287/s1, Figure S1: Phosphorescent emission spectrum of Mn-ZnS QD and Mn-ZnS QD:Ab bioconjugates. Figure S2: Absorption spectrum of AuNP and AuNP:Ab bioconjugates. Figure S3: Confocal-image obtained HR-TEM images. Figure S4: Confocal microscopy images for amplified QDs using different hydroxylamine concentrations (0.5, 1, 5, 10, 20 mM). Figure S5: Confocal microcopy images for amplified QDs using different NaAuCl_4_ concentrations (0.1, 0.2, 0.5, and 1 mM). Figure S6: Confocal microscopy images for amplified QDs using different incubation times: 10, 15, 20, and 30 min. Figure S7: NP number obtained for amplification time optimization. Table S1: Calibration points and regression obtained for immunoassay dose response curve. Figure S8: Confocal microscopy images for amplified QDs for two different synthetic samples containing BSA 1%: 0 and 10 pg·mL^−1^.
