# Supplementary material for: Catalytic Gold Deposition for Ultrasensitive Optical Immunosensing of Prostate Specific Antigen
Source: Sensors (Basel). 2020 Sep 16;20(18):5287. doi: 10.3390/s20185287 (PMC7571086; doi:10.3390/s20185287)
Supplement: Supplementary file 1 [file sensors-20-05287-s001.pdf]

Supplementary material

Catalytic Gold Deposition for Ultrasensitive of Prostate Specific Antigen

Laura Cid-Barrio, Jorge Ruiz Encinar and José Manuel Costa-Fernández*

Department of Physical and Analytical Chemistry, University of Oviedo, Av. Julián Clavería 8, 33006 Oviedo, Spain; cidlaura@uniovi.es (L.C.-B.); ruizjorge@uniovi.es (J.R.E.)

* Correspondence: jcostafe@uniovi.es

Received: 31 July 2020; Accepted: 14 September 2020; Published: date

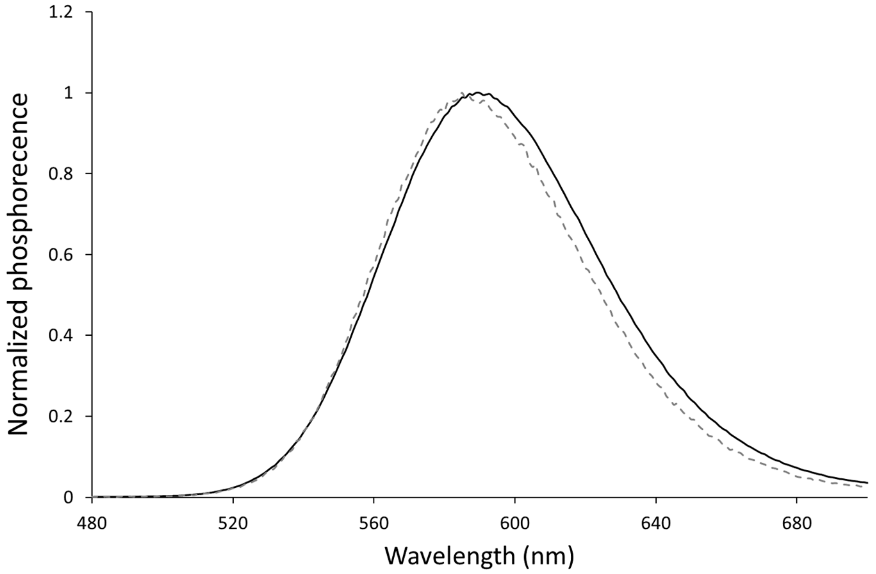

Figure 1. Phosphorescent emission intensity for Mn-ZnS QDs (black line) and Mn-ZnS QDs: antibody conjugate (dotted line).

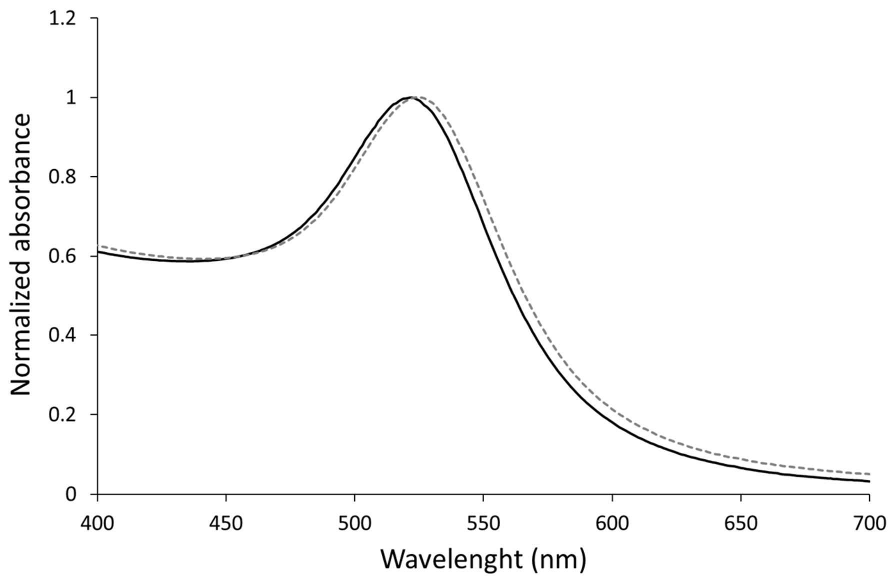

Figure S2. Absorbance spectrum of free AuNPs (black line) and AuNPs:antibody conjugate (dotted line)

(a)

(b)

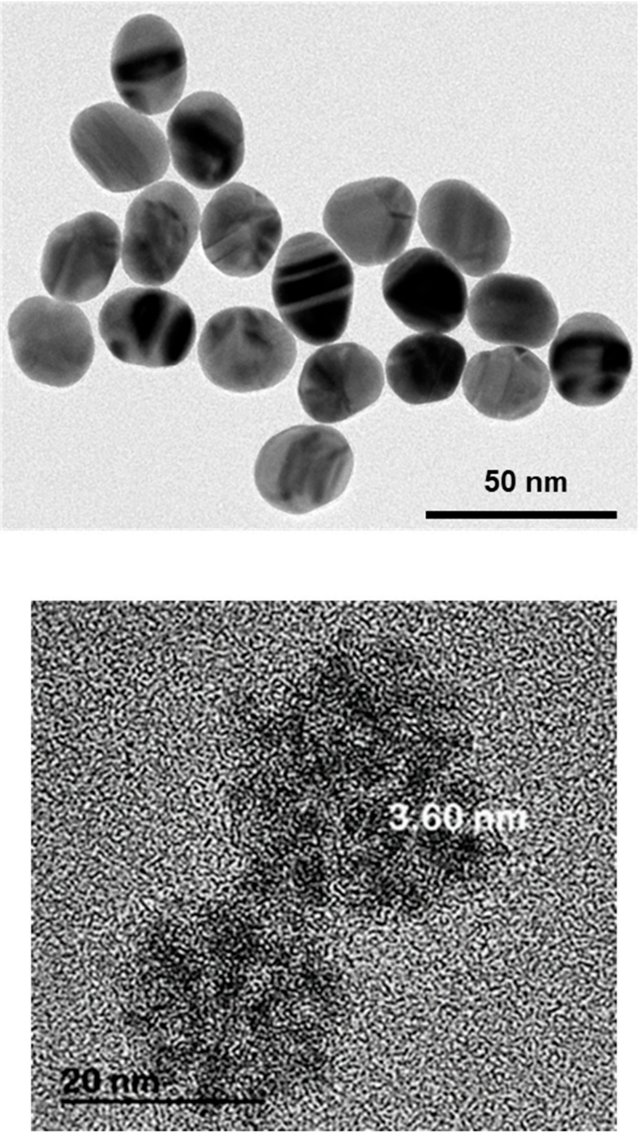

Figure S3. HR-TEM images of (**a**) AuNPs and (**b**) Mn-ZnS QDs.

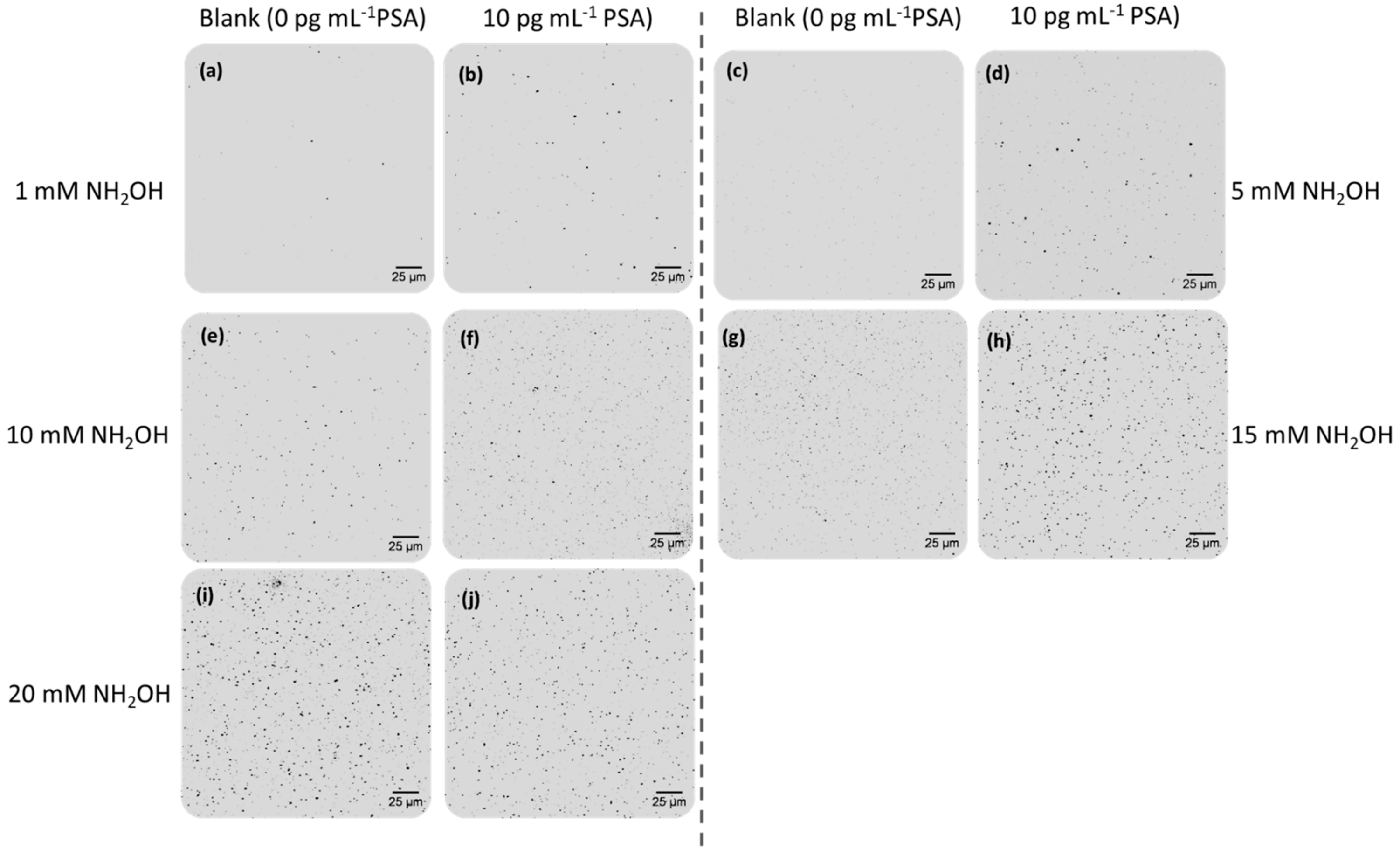

Figure S4. Confocal microscopy images of small areas (230 x 230 μm²) of the microscope slide wells obtained after the immunoassay and gold amplification performed using two PSA concentration at different concentrations of Hydroxylamine solution during amplification process using 0.5 mM NaAuCl4 concentration: (**a**) 0 pg mL^{-1} PSA, 1 mM NH_2OH; (**b**) 10 pg mL^{-1} PSA, 1 mM NH_2OH; (**c**) 0 pg mL^{-1} PSA, 5 mM NH_2OH; (**d**) 10 pg mL^{-1} PSA, 5mM NH_2OH; (**e**) 0 pg mL^{-1} PSA, 10 mM NH_2OH; (**f**) 10 pg mL^{-1} PSA, 10 mM NH_2OH; (**g**) 0 pg mL^{-1} PSA, 15 mM NH_2OH; (**h**) 10 pg mL^{-1} PSA, 15 mM NH_2OH; (**i**) 0 pg mL^{-1} PSA, 20 mM NH_2OH; (**j**) 10 pg mL^{-1} PSA, 20 mM NH_2OH.

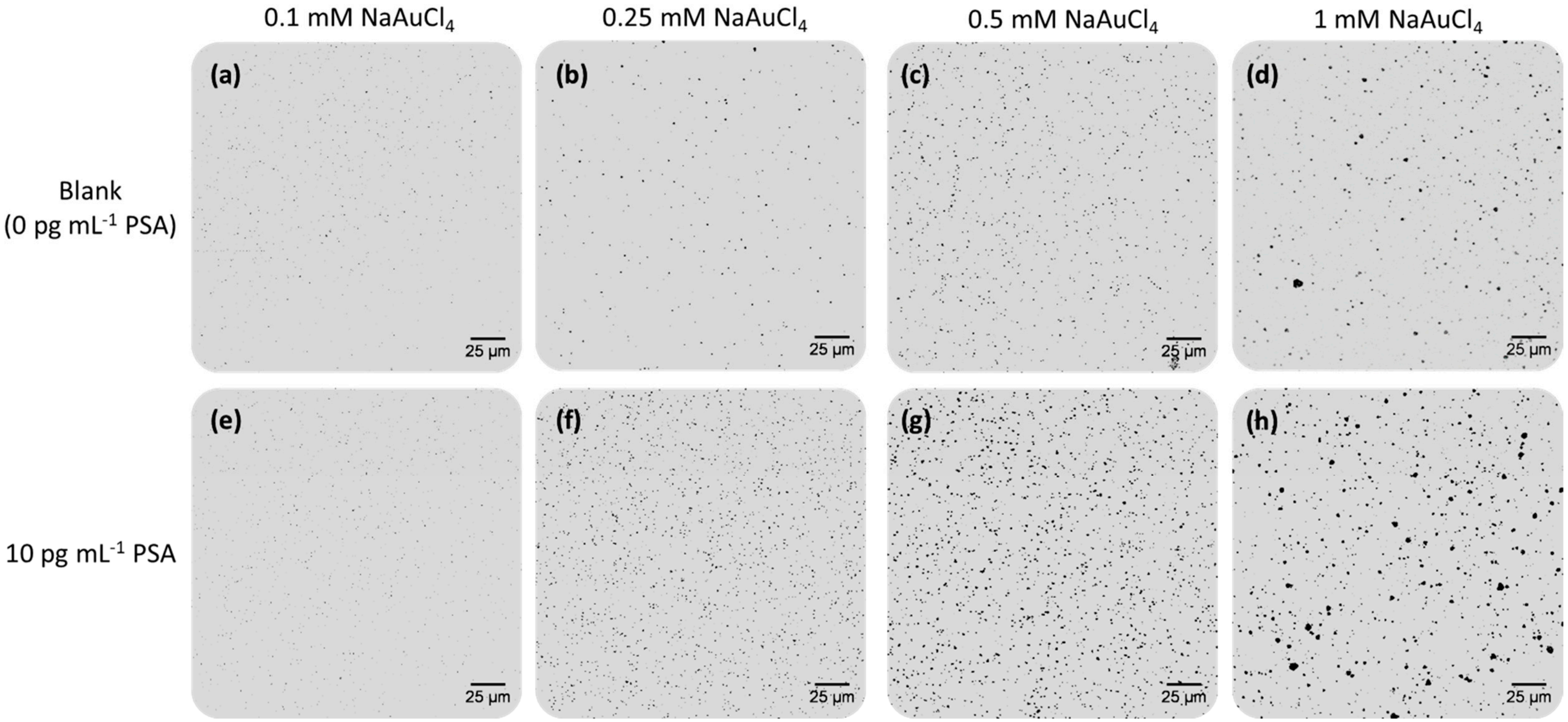

Figure S5. Confocal microscopy images of small areas (230 x 230 µm2) of the microscope slide wells obtained after the immunoassay and gold amplification performed using two PSA concentration at different concentrations of NaAuCl4 solution during the amplification process using 5 mM hydroxylamine: (**a**-**d**) blank solution (0 pg mL^{-1} PSA) incubated for amplification using 0.1, 0.25, 0.5 and 1 mM $NaAuCl_4$; respectively; (**e**-**h**) 10 pg mL^{-1} PSA incubated for amplification using 0.1, 0.25, 0.5 and 1 mM $NaAuCl_4$; respectively.

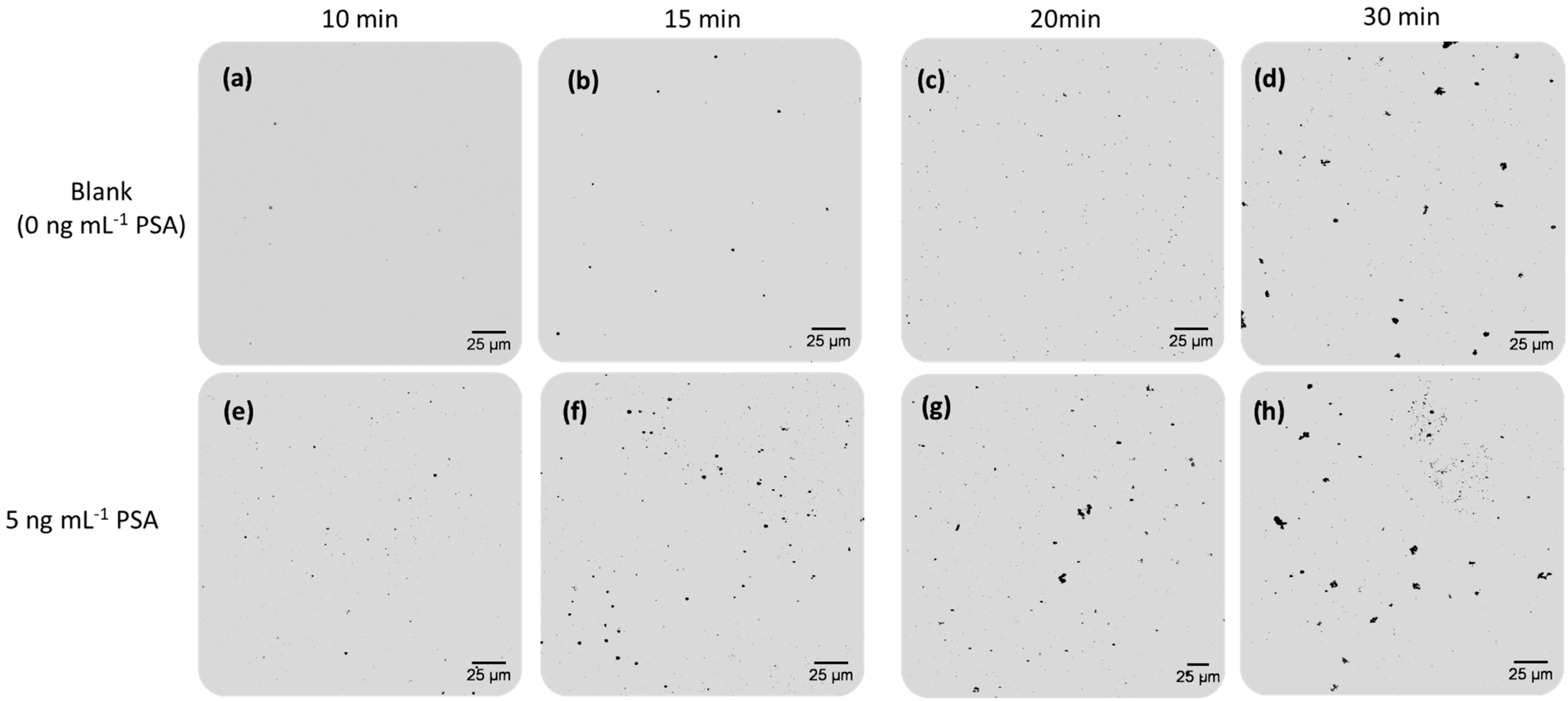

Figure S6. Confocal microscopy images of small areas (230 x 230 µm^2) of the microscope slide wells obtained after the immunoassay and gold amplification performed using two PSA concentration at different amplification times using hydroxylamine and NaAuCl4: (**a**-**d**) blank solution (0 ng mL^{-1} PSA) incubated for amplification during 10, 15, 20 and 30 min; respectively; (**e**-**h**) 5 ng mL^{-1} PSA incubated for amplification during 10. 15, 20 and 30 min; respectively.

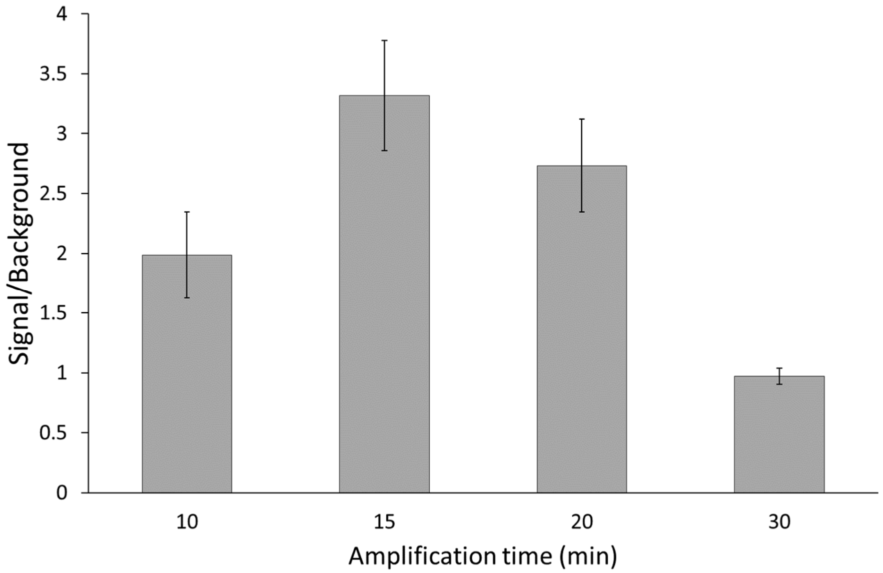

Figure S7. Signal to background signal (S/B) measured for Au-amplified Mn-ZnS QDs used as antibody tags after performing the PSA immunoassay with samples containing different PSA concentrations: blank solution (white bars) and 5 ng mL^{-1} PSA (grey bars) using different amplification times.

.

Table 1. Total particle number obtained from confocal microscopy images for each individual calibration point after de PSA QDs-based immunoassay, followed by gold deposition on the Mn-ZnS QDs. Uncertainty corresponds to 1 standard deviation (1SD). Regression values obtained for the corresponding dose response curve using net signals (particle number – particle number in black solution) are also included.

PSA concentration pg mL^{-1}	Total particle number
0	1050 ± 210
0.01	4018 ± 22
0.1	4436 ± 311
1	4808± 332
10	5294 ± 119
100	6215 ± 397

Regression curve: 228.07 Ln [PSA] + 3237.4; r^2=0.966

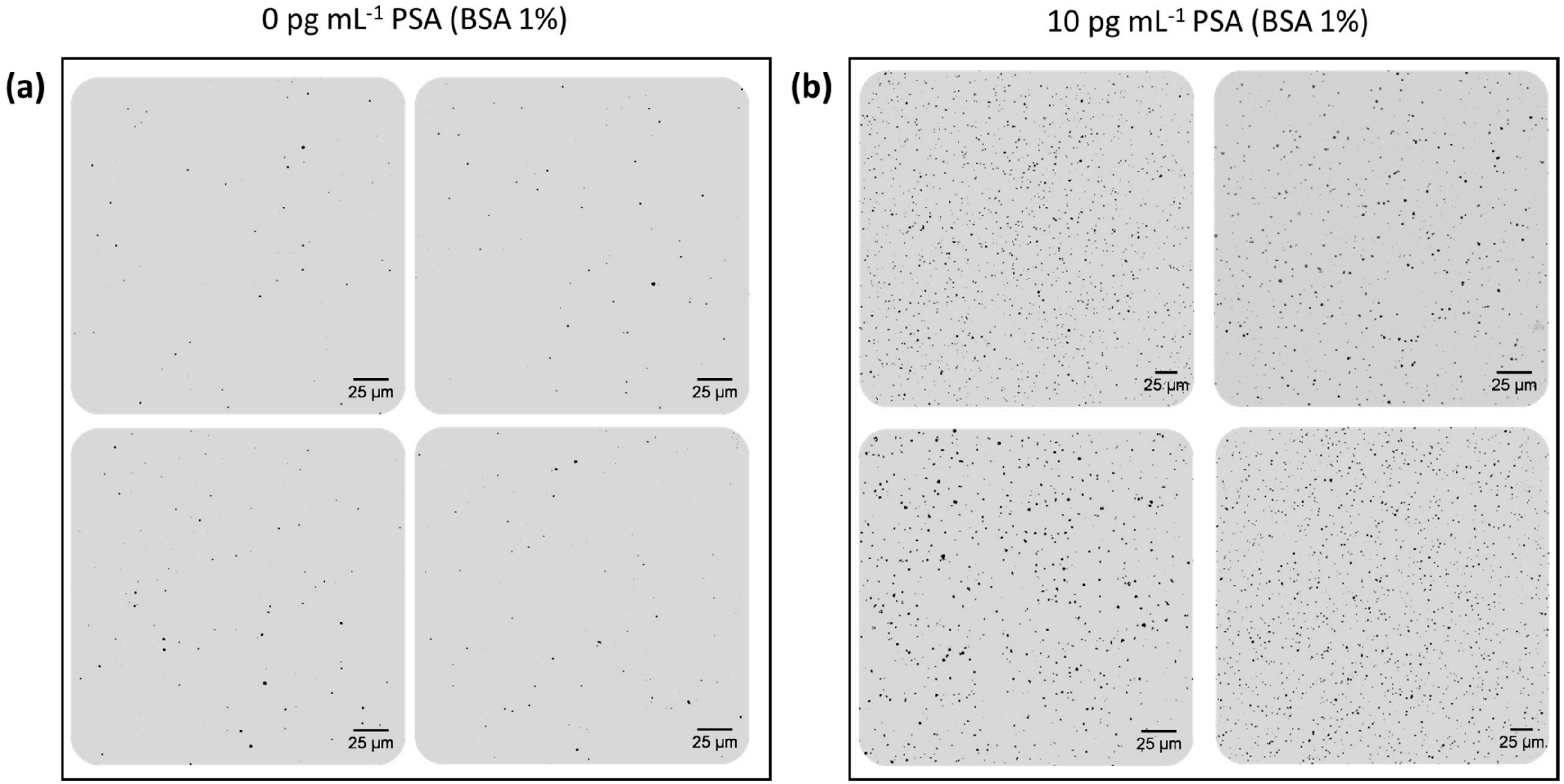

Figure S8. Confocal microscopy images of the small areas (230 x 230 µm^2) of the microscope slide wells obtained after the immunoassay and gold amplification process using optimal conditions for two different samples: (**a**) 0 pg mL^{-1} PSA and (**b**) 10 pg mL^{-1} PSA, both in presence of a high protein content (BSA 1%) Images were recorded in different zones of the same wells.
